# Effects of active commuting on cardiovascular risk factors: GISMO—a randomized controlled feasibility study

**DOI:** 10.1111/sms.13697

**Published:** 2020-08-27

**Authors:** Bernhard Reich, David Niederseer, Martin Loidl, Maria Dolores Fernandez La Puente de Battre, Valentina Alice Rossi, Bernhard Zagel, Stefano Caselli, Christian Schmied, Josef Niebauer

**Affiliations:** ^1^ Institute of Sports Medicine, Prevention and Rehabilitation and Research Institute of Molecular Sports Medicine and Rehabilitation Paracelsus Medical University Salzburg Austria; ^2^ Department of Cardiology University Heart Center Zurich University of Zurich Zürich Switzerland; ^3^ Department of Geoinformatics Paris Lodron University Salzburg Salzburg Austria; ^4^ Cardiovascular Center Zürich Hirslanden Klinik im Park Zürich Switzerland

**Keywords:** active commuting, exercise training, lifestyle modification

## Abstract

A sedentary lifestyle is a major modifiable risk factor for many chronic diseases. Lifestyle modification in order to increase exercise capacity is key in the prevention and rehabilitation of chronic diseases. This could be achieved by active commute. The aim of this study was to assess the effects of daily active commuting on physical activity (PA) and exercise capacity. Seventy‐three healthy hospital employees (age: 46 ± 9 years, 38% male), with a predominantly passive way of commuting, were randomly assigned to two parallel groups, a control group (CG, N = 22) or an intervention group (IG, N = 51), which was further split into public transportation/active commuting (IG‐PT, N = 25) and cycling (IG‐C, N = 26). Both intervention groups were asked to reach 150 min/wk of moderate‐ to vigorous‐intensity exercise during their commute for 1 year. CG maintained a passive commuting mode. All participants underwent assessment of anthropometry, risk factor stratification, and exercise capacity by a medical doctor at the Institute of Sports Medicine, Prevention and Rehabilitation. Weekly physical activity, using the International Physical Activity Questionnaire and commuting behavior, using an online diary, were used to assess physical activity. At the end of the study, the change in exercise capacity did significantly differ between IG and CG (*P* = .003, ES = 0.82). Actively covered distances through commuting significantly differed between groups (walking *P* = .026; cycling *P* < .001). Therefore, active commuting improves exercise capacity and can be recommended to the working population to increase exercise capacity**.**

## 1 INTRODUCTION

Cardiovascular disease (CVD) is the leading cause of death in the industrialized world.^1^ Risk factors for CVD are manifold. One major risk factor for CVD but also for many other non‐communicable diseases is a sedentary lifestyle. Lifestyle modification in the sense of being more active and increasing exercise capacity is key not only in the treatment of patients suffering from CVD but also in the prevention of CVD and other non‐communicable disease. Already a modest increase in physical activity (PA)^2^ leads to substantial improvement in exercise capacity and health in general.^3^, ^4^, ^5^, ^6^ Indeed, moderate‐ to vigorous‐intensity physical activity has been shown to offset the health risk of extended hours of inactivity, that is, reduces all‐cause and CVD mortality.^7^ If a certain amount of PA is not reached, exercise capacity will decrease, which is associated with increased mortality.^5^, ^6^ Despite the benefits of PA which are well understood and promoted not only by the World Health Organization (WHO)^8^ and different medical associations,^9^, ^10^, ^11^, ^12^ 31% of the global population are physically inactive^13^ and the prevalence of insufficient physical activity still increases, especially in high‐income countries.^14^ The gap between being active and inactive is only 30 minutes a day. This small amount of time is enough to reduce the risk of all‐cause mortality by 14% and results in a 3‐year longer life expectancy.^2^ The knowledge of a healthy lifestyle itself is not enough, strategies need to be found to help people to get active at a proper level, and many national health authorities have already implemented strategies.^15^, ^16^, ^17^ Indeed, active commuting has already been reported to be effective in improving exercise capacity.^18^, ^19^ Although active commuting in combination with promotion via new technologies is “en vogue” and may at least in part solve the pandemic of physical inactivity^20^ as well as possible transportation problems in cities, randomized controlled trials are still lacking to prove the effectiveness of active commuting on various risk factors and especially physical activity and exercise capacity. It was therefore the goal of this study to assess the effects on cardiovascular risk factors, especially on physical activity and exercise capacity through employer‐triggered interventions to meet the World Health Organization (WHO) recommendations of moderately exercising 150 minutes a week^8^ while commuting. We hypothesized that implementing the WHO recommendation for exercise into the daily commute would increase physical activity and exercise capacity.

## 2 METHODS

This study utilized a 2:1 randomized, control group design consisting of an intervention group (with two different ways to promote active commuting according to the distance of the participant and the working place) and a control group, to develop a framework for future studies on active commuting.^21^

### 2.1 Study approval and design

The GISMO (Geographical Information Support for healthy MObility) study complied with the Declaration of Helsinki and its current amendments and was approved by the Ethical Committee of the Paris Lodron University Salzburg, Austria (EK‐GZ: 43/2016). The trial is registered at ClinicalTrials.gov (NCT03098719). All participants provided written informed consent before study inclusion.

### 2.2 Subjects

**FIGURE 1:**
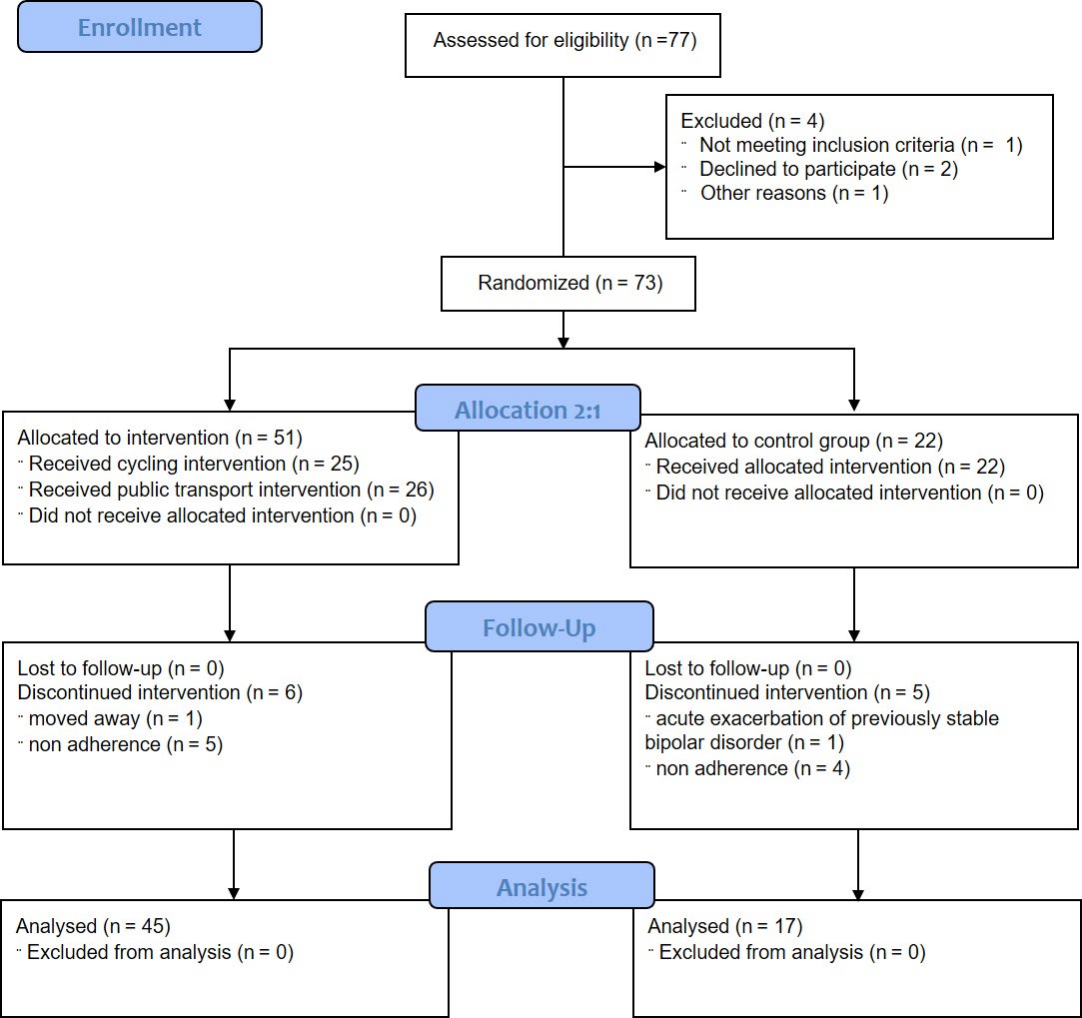
Flowchart of the study

Employees of the hospitals of the State of Salzburg (Salzburger Landeskliniken, SALK, Austria) between 18 and 75 years who were able to properly understand the protocol in German, motivated to change their commuting habits, and willing to take part in the study over 1 year were considered for inclusion. Subjects with known physical or psychiatric conditions precluding regular physical exercise were excluded. In particular, the following conditions were considered not to be compatible with the study: simultaneous or <4‐week participation in other clinical studies, pregnancy or lactation, known musculo‐skeletal conditions impairing mobility, subjects with known depression or other psychiatric disorders in the last 5 years prior to enrollment (ie, panic, obsessive‐compulsive disorder, schizophrenia, organic mental disorder, psychotic, phobic conditions), osteoporosis in need of treatment, severe general diseases (neoplasia, tuberculosis, heart failure), chronic infections, and subjects with active alcohol or drug abuse or addiction. Subjects interested in our study contacted our research administrator and were considered if they were motivated to commute actively using public transport as well as cycling or walking. An analysis of the screening process of this study is provided elsewhere,^22^ and a flowchart of the study is shown in Figure 1.

### 2.3 Randomization

Randomization was performed with a 2:1 stratum to an intervention group (IG) and a control group (CG). Eighty envelopes were filled with 54 lots for IG and 26 for CG. At study entry, each participant picked a sealed envelope showing him his group assignment. Unused envelopes were discarded (n = 7).

### 2.4 Data acquisition

Data were collected at the beginning and end of the study from adherent participants to allow assessment of the effects of regular active commuting on cardiovascular risk profile, daily activity, physical performance, quality of life, mobility behavior, and body composition. Non‐adherent participants did not continue to commute actively. All investigations were pre‐specified in the ethics protocol and on ClinicalTrials.gov.

At all time points, subjective effects of active commuting were analyzed by questionnaires: SF‐36 questionnaire, to explore the quality of life; IPAQ (International Physical Activity Questionnaire), to estimate the amount of daily physical activity; and a questionnaire about mobility (see Appendix S1). The Heart Score of the European Society of Cardiology was utilized to analyze cardiovascular risk factors.^23^ Blood tests including lipid values and HbA1c measurements were performed in fasting condition after 10 minutes of resting and were immediately handed over to the laboratory for further analysis. An electrocardiogram (ECG) was performed using AmedTec ECGpro software and hardware. Anthropometric measurements included height, weight, body mass index, abdominal circumference measured above the hips, and analysis of body fat composition using the 4‐point measurements of skinfold thickness, with the Harpenden calliper^24^ at the following landmarks: (a) m. triceps brachii, (b) m. biceps brachii, (c) subscapularly (under the angulus inferior), and (d) abdominal (pinch placed vertically 5cm adjacent to the umbilicus to the right side); for each position, the average of three measurements was taken.

In accordance with respective guidelines, physical examination, personal history, and a 12‐lead resting ECG and a spirometry at rest were performed in every subject before ergometry by a medical doctor at the Institute of Sports Medicine, Prevention and Rehabilitation, Salzburg. The institute has a volume of approximately 2500 patients per year. Ergometries were performed to assess the primary outcome of the study, that is, exercise capacity. Exercise protocols were in accordance with the Austrian guidelines of exercise testing^25^ and kept identical for male and female participants during all tests to permit accurate comparison. Male participants started a ramp protocol at 60 W, and performance was increased every minute by 20 W; female participants started at 40 W with a 15W increase every minute. During ergometry, a 12‐lead electrocardiogram (ECG) continuously monitored heart rate; blood pressure was measured manually at rest and subsequently during ergometry. In all participants, ergometries were stopped when volitional exhaustion or pre‐specified criteria for termination (listed elsewhere^25^) were reached.

Mobility characteristics were recorded by mobility diaries, which were validated and corrected with the help of GPS‐equipped fitness watches (Polar M200). Participants were asked to wear these devices during 2 weeks at the beginning and 2 weeks toward the end of the intervention period. With this combined approach, the validity of the mobility documentation could have been increased substantially compared to self‐reported approaches.^26^

### 2.5 Intervention

Intervention group A (IG‐PT) included participants who lived further away, and in addition to using public transportation, they were expected to get off at least one stop further away from work and home as they usually would. Also, they were asked to walk the remaining distance. Alternatively, participants could also use a bicycle to cover this distance. As part of this intervention, tickets for the study duration were provided as an incentive to switch from cars to public transportation.

Intervention group B (IG‐C) included those participants who lived ≤10 km from work. These participants were expected to change from car to cycling and/or walking for at least 50% of the total commuting distance. In case of bad weather or exceptional circumstances, it was allowed to use the car or public transportation.

Control group: Participants allocated to the control group were asked to continue to commute as usual. A total of 20‐25 participants in each group were needed to complete the study.

Participants reported their commuting details, which were periodically verified by GPS tracking.

However, both groups were asked to reach the WHO recommendation of 150 minutes of at least moderate exercise intensity during their commute for 1 year, whereas CG was asked not to change their mode of commuting. Each group received incentives to keep them motivated, and participants were randomized to the intervention group to refrain from using the car to commute; for example, all participants had the opportunity to have their bicycles repaired free of charge, received rain gear, and other useful items.^22^

### 2.6 Statistical analysis

All variables were tested for normal distribution by the Kolmogorov‐Smirnov or Shapiro‐Wilk test. As all variables were normally distributed, data are shown as mean and 95% confidence intervals (CI), or standard deviation (STD), mean, and 95% CI of the delta between the beginning and end of the study. Differences in baseline characteristics between groups were assessed by independent *t* tests and chi‐square test as appropriate. Per‐protocol analysis was performed with ANOVA for PA and ANCOVA accounting for baseline values and sex, to assess differences between groups. Subsequent post‐hoc analysis was tested with Bonferroni correction. A two‐sided *P*‐value of .05 was considered to be statistically significant. Effect size (ES) was calculated as the mean of IG – mean of CG divided by the standard deviation of the pooled values of all participants. All statistical analyses were performed with IBM SPSS Statistics version 21 (SPSS, Inc). No a priori sample size calculation was performed since the sample size was defined by the willingness of includable hospital staff to participate in the study. However, great effort was undertaken to recruit as many participants as highlighted by the recruitment process provided elsewhere.^22^

## 3 RESULTS

Seventy‐seven healthy hospital employees were assessed for eligibility; four subjects were excluded or declined to participate, and 11 dropped out during the intervention period. Finally, 17 patients were analyzed in CG, 23 participants in IG‐PT, and 22 participants in IG‐C. Participants worked in all hospital areas (21 nurses [34%], nine physicians [15%], 20 working in administration [32%], and 12 in various other professions [19%]). Baseline characteristics are shown in Table 1. Distances covered through active commuting significantly differed between groups, and an increase in PA, measured with IPAQ, did not reach the level of significance (Table 2).

**TABLE 1:** Baseline characteristics of the control group (CG), public transportation/active commuting (IG‐PT), and cycling (IG‐C)

	ALL (N = 62)	CG (N = 17)	IG‐PT (N = 23)	IG‐C (N = 22)
Age (y)	46 [9]	45 [10]	47 [9]	47 [8]
Men (%)	36	29	30	45
Anthropometrics				
Height (m)	1.71 [0.1]	1.71 [0.1]	1.69 [0.1]	1.74 [0.1]
Weight (kg)	76.5 [16.5]	77.7 [20.3]	73.5 [14.5]	78.7 [15.4]
BMI (kg/m^2^)	26.0 [4.5]	26.4 [5.5]	25.8 [4.4]	26.0 [4.0]
Waist circ. (cm)	91.1 [13.2]	92.6 [16.3]	89.1 [11.5]	91.9 [12.5]
Hip circ. (cm)	103.2 [11.3]	101.8 [13.2]	103.3 [12.6]	104.1 [8.4]
Waist‐to‐hip ratio	0.9 [0.1]	0.9 [0.1]	0.9 [0.1]	0.9 [0.1]
Body fat (%)	34.6 [0.8]	35.0 [9.1]	35.0 [7.6]	34.0 [7.9]
Spirometry				
FVC (L)	4.1 [1.0]	4.0 [0.7]	4.0 [1.0]	4.3 [1.1]
FEV1 (L)	3.2 [0.8]	3.1 [0.7]	3.1 [0.8]	3.4 [1.0]
PEF (L)	7.8 [2.2]	7.6 [1.9]	7.3 [2.0]	8.4 [2.5]
FEV1/FVC	0.8 [0.1]	0.8 [0.1]	0.8 [0.1]	0.8 [0.1]
Lipid and glucose metabolism				
TRI (mg/dL)	90.9 [43.4]	86.0 [51.4]	98.0 [40.5]	87.2 [40.5]
CHOL (mg/dL)	205.3 [34.3]	200.2 [38.6]	211.8 [35.8]	202.4 [29.4
HDL (mg/dL)	74.3 [23.8]	83.7 [34.6]	68.4 [17.4]	73.1 [17.6]
LDL (mg/dL)	113.4 [30.7}	101.1 [21.8]	123.9 [35.9]	112.0 [27.9]
LDL/HDL ratio	1.7 [0.8]	1.4 [0.6]	2.0 [0.9]	1.6 [0.6]
HbA1c (%)	5.3 [0.3]	5.3 [3.4]	5.3 [0.2]	5.4 [0.3]
GLU (mg/dL)	78.0 [13.0]	78.2 [13.3]	75.3 [10.3]	80.7 [15.1]
Cardiovascular risk scores				
FRS score	2.4 [3.0]	3.0 [4.4]	2.2 [2.4]	2.1 [2.1]
HS score	0.5 [0.7]	0.6 [0.9]	0.5 [0.7]	0.5 [0.4]
Blood pressure				
RR_sys_ (mm Hg)	114 [13]	114 [14]	112 [10]	117 [15]
RR_dia_ (mm Hg)	72 [11]	71 [9]	71 [9]	75 [13]
Exercise capacity				
*P*_max_ (W)	221 [70]	210 [56]	203 [58]	248 [85]
P_maxrel_ (%)	144 [25]	142 [25]	138 [22]	152 [27]
HR_max_ (bpm)	171 [13]	171 [14]	171 [12]	172 [14]
HR_rest_ (bpm)	63 [9]	66 [9]	63 [5]	62 [11]
BORG	19.1 [0.4]	19.1 [0.3]	19.0 [0.4]	19.1 [0.4]
IPAQ				
Total PA (METmin^‐1^)	4166 [4431]	5251 [5076]	3143 [3725]	4398 [4550]
Walk (METmin^‐1^)	1370 [2115]	1427 [1848]	1053 [1262]	1657 [2919]
Mod (METmin^‐1^)	1015 [1631]	1121 [2251]	540 [738]	1428 [1691]
Vig (METmin^‐1^)	1782 [2391]	2703 [3068]	1550 [2612]	1313 [1141]
Sit (min)	377 [167]	346 [151]	411 [205]	367 [133]

Note

Data are shown as mean [SD].

Abbreviations: BMI, body mass index; BORG, rating of perceived exertion; CHOL, total cholesterol; FEV1, forced expiratory volume in the 1^st^ second; FRS score, Framingham risk score; FVC, forced vital capacity; GLU, fasting blood glucose; HbA1c, hemoglobin A1c; HDL, HDL cholesterol; Hip circ., hip circumference; HR_max_, maximal heart rate at ergometry; HR_rest_, resting heart rate; HS score, Heart Score of the European Society of Cardiology (ESC); IPAQ, International Physical Activity Questionnaire; LDL, LDL cholesterol; Mod, moderate‐intensity activities within the last 7 d; N, number of participants; PEF, peak expiratory flow; *P*_max_, exercise capacity; P_maxrel_, age and weight‐normalized exercise capacity; PT, public transportation; RR_dia_, diastolic blood pressure; RR_sys_, systolic blood pressure; Sit, sitting time during the last 7 d; Total PA, total physical activities during the last 7 d; TRI, triglycerides; Vig, vigorous‐intensity activities within the last 7 d; Waist circ., waist circumference; Walk, walking within the last 7 d.

**TABLE 2:** Change in exercise capacity, IPAQ and covered distances of study participants of the control group (CG), public transportation/active commuting (IG‐PT), and cycling (IG‐C)

	CG (N = 17)	IG‐PT (N = 23)	ES	IG‐C (N = 22)	ES	*P*
*P*_max_ (W)	−8 [−19, 3]	13 [4, 23]	0.78	14 [1, 28]	0.82	.013
P_maxrel_ (%)	−6 [−13, 0]	9 [3, 15]	0.92	10 [1, 18]	0.97	.003
HR_max_ (bpm)	−4 [−10, 2]	3 [0, 7]	0.61	2 [−4, 8]	0.52	.111
BORG	−0.1 [0, 0]	0.0 [0, 0.1]	0.35	−0.1 [−0.2, 0]	0.00	.204
IPAQ						
Total PA (METmin^−1^)	717 [−1717, 3150]	3200 [1546, 4853]	0.50	3466 [855, 6078]	0.57	.295
Walk (METmin^−1^)	221 [−633, 1074]	814 [−85, 1712]	0.23	712 [−881, 2305]	0.19	.400
Mod (METmin^−1^)	674 [−566, 1913]	1117 [206, 2028]	0.05	453 [−129, 1034]	−0.12	.944
Vig (METmin^−1^)	−178 [−1538, 1183]	1269 [170, 2368]	0.46	2302 [583, 4020]	0.78	.129
Sitting (min)	23 [−80, 126]	−61 [−171, 48]	0.32	−52 [−101, −3]	−0.39	.442
Covered distances						***P*_ANOVA_**
Walking (km)	110 [19, 202]	305 [203, 407]	0.91	65 [12, 119]	−0.21	<.001
Cycling (km)	35 [0, 73]	450 [185, 716]	0.39	1673 [1159, 2187]	1.56	<.001
PT (km)	273 [0, 681]	1861 [357, 3366]	0.54	1248 [0, 2642]	0.33	.230
Car (km)	2371 [30, 4711]	299 [73, 526]	−0.90	322 [29, 616]	−0.89	.016

Note

Data are mean differences of end‐beginning [95% CI].

Abbreviations: BORG, rating of perceived exertion; ES, effect size between CG and IG‐PT/IG‐C; HR_max_, maximal heart rate at ergometry; Mod, moderate‐intensity activities within the last 7 d; *P*, ANCOVA adjusted for baseline and sex; *P*_max_, exercise capacity; P_maxrel_, age and weight‐normalized exercise capacity; Sit, sitting time during the last 7 d; Total PA, total physical activities during the last 7 d; Vig, vigorous‐intensity activities within the last 7 d; Walk, walking within the last 7 d.

**FIGURE 2:**
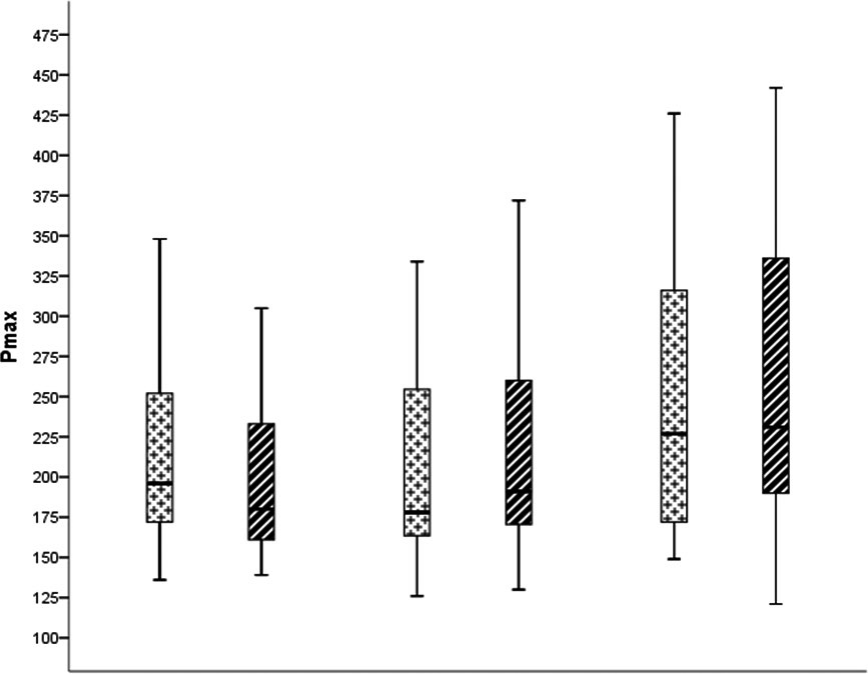
Exercise capacity at the beginning and end of study participants of the control group (CG), public transportation/active commuting (IG‐PT), and cycling (IG‐C). *P < .05 *P*_max_ CG vs. IG‐PT vs IG‐C. CG, control group; IG‐C, intervention group cycling; IG‐PT, intervention group public transportation; *P*_max_, exercise capacity

Changes in exercise capacity during the study significantly differed between IG (14 W [95% CI: 6, 22] *P* = .003, ES = 0.82) and CG (−8 W [95% CI: −19, 3]). Exercise capacity at the end of the study significantly differed between IG (239 W [95% CI: 213, 264] *P* = .003, ES = 0.47) and CG (202 W [95% CI: 175, 229]) as well as between IG‐PT and IG‐C and CG (*P* = .013). A Bonferroni post‐hoc test revealed that exercise capacity was statistically different compared to IG‐PT (216 W [95% CI: 186, 247] *P* = .032, ES = 0.18) and IG‐C (262 W [95% CI: 220, 305] *P* = .025, ES = 0.76) and CG (Figure 2; Table 2).

Changes in *P*_maxrel_ during the study significantly differed between IG (9% [95% CI: 5, 14] *P* < .001, ES = 0.95) and CG (−6% [95% CI: −13, 0]). *P*_maxrel_ at the end of the study significantly differed between IG (154% [95% CI: 145, 163], *P* < .001, ES = 0.60) and CG (136% [95% CI: 123, 148]) as well as between IG‐PT and IG‐C and CG (*P* = .003). A Bonferroni post‐hoc test revealed that *P*_maxrel_ was statistically different compared to IG‐PT (147% [95% CI: 123, 148], *P* = .003, ES = 0.37) and IG‐C (162% [95% CI: 147, 176], *P* = .025, ES = 0.86) and CG (Figure 2; Table 2).

Neither HR_max_ nor the BORG scale did significantly differ between groups (*P* = .111 and *P* = .204, respectively) (Figure 2; Table 2).

## 4 DISCUSSION

The results of the GISMO study show that the change to active commuting increases PA and exercise capacity. Both intervention groups, but not CG, which did not commute actively, significantly improved exercise capacity. PA, in the sense of active commuting, did significantly differ between groups. Based on our results, we can recommend active commuting to the working population to counteract physical inactivity and to increase exercise capacity.

As exercise capacity is one of the best prognostic factors for cardiovascular risk as well as all‐cause morbidity and mortality, generally one benefits from an increased exercise capacity.^27^, ^28^ For that reason, medical associations and public authorities do recommend to stay or become physically active. People are aware of this fact as they rate the aspect of their lifestyle that they needed to change the most, “lack of exercise” as the highest.^29^

Not surprisingly, it is the sedentary person that benefits most from starting an exercise‐training program, as already modest improvements in exercise capacity are associated with lower cardiovascular event rates.^30^ In our study, exercise capacity was improved by 7% in the IG‐PT and by 6% in the IG‐C, which is in accordance with shorter^31^ as well as longer^32^ interventions on active commuting. More interventions are addressed in a systematic review^33^ of intervention studies with active commuting as the primary intervention. Based on these studies, we can speculate that active commuting is also associated with lover cardiovascular event rates.^34^

Higher improvements in exercise capacity through commuting were reported by Hendriksen et al^35^ Fujimoto et al^36^ reported a 19% increase in exercise capacity in previously sedentary, healthy seniors, enrolled in a 1‐year training program. Also Howden et al^37^ documented in sedentary middle‐aged participants a 19% improvement in exercise capacity after 2 years of exercise training. Increase in exercise capacity of such magnitude should not be expected if participants, who previously commuted passively to work, suddenly adopt an active mode of transportation, may it be by walking or cycling. To reach comparable improvement during studies of active commuting that are comparable to training studies, recommendations on duration and intensity have to be implemented. Blond et al^32^ found that also in active commuters, gains in cardiorespiratory fitness increase with exercise intensity. As Blond et al reported that the increase in physical fitness was steeper during the first than during the second 3 months, this nicely illustrates the need to readjust exercise intensity and duration on a regular basis.

We assumed that weather would play an important role in activity behavior. We expected that especially cold and snowy winter conditions could be a barrier to physically active commuting. Surprisingly, this was not the case. Participants were active even during wintertime and did not use their cars more often than during summer. A detailed analysis on the seasonal aspects of active commuting in the GISMO study is provided elsewhere.^26^ This is in contrast to Stigell et al^38^ who found lower cycling frequencies during winter than during summer. Walking commuters in contrast maintained active commuting also during winter. In our study, commuters continued to walk and cycle during winter, which possibly consolidated exercise capacity during this 1‐year study.

Not only climate conditions, but probably even more importantly the availability and quality of sidewalks and bike paths might be barriers for active commuters. Panter et al^39^ reported that commuters who find a supportive environment for walking and cycling were more likely to incorporate walking or cycling into commuting. This was also true for people who had to pay for parking. Also Giles‐Corti et al^40^ as well as Sallis et al^41^ called for better planning of cities to make active transportation but also physical activity, in general, more attractive. Adequate infrastructure is fundamental for physically active commuting.^42^ On the other hand, one might argue that it is impossible for many employees to commute actively, as distances are too long. In this case, we recommend intermodal strategies with active parts in the trip chain.

The integration of exercise into one's daily routine is one way to overcome sedentary behavior. Thirty minutes of active commuting should be possible for most people. Our results show that this is indeed feasible and that it significantly improves exercise capacity without needing to spend extra time. Time could even be saved as there is by far less time needed than it would take to go to the gym or take lessons with a personal coach or in a sports club. Interestingly, total time spent for commuting was not significantly higher during active as compared to passive commute.

In conclusion, lifestyle modification for prevention but also therapy is key in many chronic diseases. Recommendations of different medical associations and public health institutions are similar but ineffective as activity levels in the industrialized world decrease.^14^ Effective and time‐efficient strategies need to be developed to increase activity levels and exercise capacity. Active commuting is one option to increase PA and to improve exercise capacity without spending extra time.

The strength of this study lies in the fact that it is a RCT in active commuting and that the collaboration between the medical field and geoinformatics fostered complementary insights and results. A limitation of our study is that on average, participants were at a low risk for lifestyle‐related diseases. Also, they were all recruited from the same workplace easily accessible by public transportation and by bike. Generalizability of our results is therefore difficult. There was no sample size calculation performed, since the funder had pre‐defined the sizing of the trial. Criteria for relevant change or non‐adherence were not defined prior to the beginning of the study. Even though there were only few dropouts, our “per‐protocol analyses” might have led to an overestimation of results. The separation of IG into IG‐PT and IG‐C by searching for the best possible commute may have biased the randomization.

## 5 PERSPECTIVES

In this randomized controlled study investigating the effects of active commuting, we can show beneficial effects on physical activity and exercise capacity. Based on our results as well as on other studies,^32^, ^43^ active commuting may be recommended to policymakers, employers, employees, co‐workers, or patients in order to improve physical activity and exercise capacity and to possibly even reduce mortality in the working force. On the one hand, much needs to be done with respect to infrastructure to make active commuting more attractive, as adequate infrastructure is fundamental for active commuting.^42^ Only then an increasing number of employees can benefit from its health effects. This will not only make people healthier but will generate multiple side effects, such as reducing the number of cars.^40^ On the other hand, more studies have to be performed to individualize recommendations on active commuting as well as duration and intensity of the commute. As most of the studies are observational studies, better improvements in exercise capacity and risk factors are potentially possible.

## DISCLOSURES

None.

## Supporting information

Appendix S1
