# Supplementary material for: Effects of active commuting on cardiovascular risk factors: GISMO—a randomized controlled feasibility study
Source: Scand J Med Sci Sports. 2020 Aug 27;30(Suppl 1):15–23. doi: 10.1111/sms.13697 (PMC7496369; doi:10.1111/sms.13697)
Supplement: Supplementary file 1 — Appendix S1 [file SMS-30-15-s001.docx]

| id |  |
| --- | --- |
| Abgeschlossen |  |
| Letzte Seite |  |
| Start-Sprache |  |
| Zugangsschlüssel |  |
| Datum gestartet |  |
| Datum letzte Aktivität |  |
| IP-Adresse |  |
| Bitte kreuzen Sie den heutigen Arbeitstag an: | |
| Mit welchem Verkehrsmittel bzw. mit welchen Verkehrsmitteln sind Sie heute zur Arbeit gelangt -  Bitte alle benutzten Verkehrsmittel angeben! [zu Fuß] | ja/nein |
| Mit welchem Verkehrsmittel bzw. mit welchen Verkehrsmitteln sind Sie heute zur Arbeit gelang -  Bitte alle benutzten Verkehrsmittel angeben! [Rad] | ja/nein |
| Mit welchem Verkehrsmittel bzw. mit welchen Verkehrsmitteln sind Sie heute zur Arbeit gelang -  Bitte alle benutzten Verkehrsmittel angeben! [E-Bike] | ja/nein |
| Mit welchem Verkehrsmittel bzw. mit welchen Verkehrsmitteln sind Sie heute zur Arbeit gelang -  Bitte alle benutzten Verkehrsmittel angeben! [Moped / Motorrad] | ja/nein |
| Mit welchem Verkehrsmittel bzw. mit welchen Verkehrsmitteln sind Sie heute zur Arbeit gelang -  Bitte alle benutzten Verkehrsmittel angeben! [Pkw als FahrerIn] | ja/nein |
| Mit welchem Verkehrsmittel bzw. mit welchen Verkehrsmitteln sind Sie heute zur Arbeit gelang -  Bitte alle benutzten Verkehrsmittel angeben! [Pkw als MitfahrerIn] | ja/nein |
| Mit welchem Verkehrsmittel bzw. mit welchen Verkehrsmitteln sind Sie heute zur Arbeit gelang -  Bitte alle benutzten Verkehrsmittel angeben! [O-Bus] | ja/nein |
| Mit welchem Verkehrsmittel bzw. mit welchen Verkehrsmitteln sind Sie heute zur Arbeit gelang -  Bitte alle benutzten Verkehrsmittel angeben! [Regionalbus] | ja/nein |
| Mit welchem Verkehrsmittel bzw. mit welchen Verkehrsmitteln sind Sie heute zur Arbeit gelang -  Bitte alle benutzten Verkehrsmittel angeben! [Bahn (inkl. Schnell-/Lokalbahn)] | ja/nein |
| Mit welchem Verkehrsmittel bzw. mit welchen Verkehrsmitteln sind Sie heute zur Arbeit gelang -  Bitte alle benutzten Verkehrsmittel angeben! [Sonstiges (z.B. Taxi)] | ja/nein |
| Ist die von Ihnen, für den heutigen Tag, angegebene  Verkehrsmittelwahl für Ihren Arbeitsweg typisch: | |
| Welches Verkehrsmittel / Welche Verkehrsmittel benutzen Sie üblicherweise für den Weg zur Arbeit -  Bitte alle benutzten Verkehrsmittel angeben! [zu Fuß] | ja/nein |
| Welches Verkehrsmittel / Welche Verkehrsmittel benutzen Sie üblicherweise für den Weg zur Arbeit -  Bitte alle benutzten Verkehrsmittel angeben! [Rad] | ja/nein |
| Welches Verkehrsmittel / Welche Verkehrsmittel benutzen Sie üblicherweise für den Weg zur Arbeit -  Bitte alle benutzten Verkehrsmittel angeben! [E-Bike] | ja/nein |
| Welches Verkehrsmittel / Welche Verkehrsmittel benutzen Sie üblicherweise für den Weg zur Arbeit -  Bitte alle benutzten Verkehrsmittel angeben! [Moped / Motorrad] | ja/nein |
| Welches Verkehrsmittel / Welche Verkehrsmittel benutzen Sie üblicherweise für den Weg zur Arbeit -  Bitte alle benutzten Verkehrsmittel angeben! [Pkw als FahrerIn] | ja/nein |
| Welches Verkehrsmittel / Welche Verkehrsmittel benutzen Sie üblicherweise für den Weg zur Arbeit -  Bitte alle benutzten Verkehrsmittel angeben! [Pkw als MitfahrerIn] | ja/nein |
| Welches Verkehrsmittel / Welche Verkehrsmittel benutzen Sie üblicherweise für den Weg zur Arbeit -  Bitte alle benutzten Verkehrsmittel angeben! [O-Bus] | ja/nein |
| Welches Verkehrsmittel / Welche Verkehrsmittel benutzen Sie üblicherweise für den Weg zur Arbeit -  Bitte alle benutzten Verkehrsmittel angeben! [Regionalbus] | ja/nein |
| Welches Verkehrsmittel / Welche Verkehrsmittel benutzen Sie üblicherweise für den Weg zur Arbeit –  Bitte alle benutzten Verkehrsmittel angeben! [Bahn (inkl. Schnell-/Lokalbahn)] | ja/nein |
| Welches Verkehrsmittel / Welche Verkehrsmittel benutzen Sie üblicherweise für den Weg zur Arbeit -  Bitte alle benutzten Verkehrsmittel angeben! [Sonstiges (z.B. Taxi)] | ja/nein |
| Wie lang ist die Strecke zwischen Ihrer Wohnung und Ihrem Arbeitsort - [ca. in Kilometer ...] |  |
| Wo parken Sie Ihr Fahrzeug (Pkw oder Moped/Motorrad) üblicherweise am Arbeitsort | Fixe Parkmöglichkeit/ Öffentlicher Straßenraum |
| Wo parken Sie Ihr Fahrzeug (Pkw oder Moped/Motorrad) üblicherweise am Arbeitsort [Sonstiges] |  |
| Wie wichtig sind Ihnen die folgenden Kriterien für Ihre persönliche Verkehrsmittelwahl auf dem Arbeitsweg -  Bitte kreuzen Sie in jeder Zeile ein Kästchen an. [Unabhängigkeit] | wichtig/ eher wichtig/ unwichtig/ eher unwichtig |
| Wie wichtig sind Ihnen die folgenden Kriterien für Ihre persönliche Verkehrsmittelwahl auf dem Arbeitsweg -  Bitte kreuzen Sie in jeder Zeile ein Kästchen an. [Zeitersparnis] | wichtig/ eher wichtig/ unwichtig/ eher unwichtig |
| Wie wichtig sind Ihnen die folgenden Kriterien für Ihre persönliche Verkehrsmittelwahl auf dem Arbeitsweg -  Bitte kreuzen Sie in jeder Zeile ein Kästchen an. [Kostenersparnis] | wichtig/ eher wichtig/ unwichtig/ eher unwichtig |
| Wie wichtig sind Ihnen die folgenden Kriterien für Ihre persönliche Verkehrsmittelwahl auf dem Arbeitsweg -  Bitte kreuzen Sie in jeder Zeile ein Kästchen an. [Bequemlichkeit/ Komfort] | wichtig/ eher wichtig/ unwichtig/ eher unwichtig |
| Wie wichtig sind Ihnen die folgenden Kriterien für Ihre persönliche Verkehrsmittelwahl auf dem Arbeitsweg -  Bitte kreuzen Sie in jeder Zeile ein Kästchen an. [Umweltschutz] | wichtig/ eher wichtig/ unwichtig/ eher unwichtig |
| Wie wichtig sind Ihnen die folgenden Kriterien für Ihre persönliche Verkehrsmittelwahl auf dem Arbeitsweg -  Bitte kreuzen Sie in jeder Zeile ein Kästchen an. [Private Erledigungen (z.B. Kinder in die Schule bringen)] | wichtig/ eher wichtig/ unwichtig/ eher unwichtig |
| Wie wichtig sind Ihnen die folgenden Kriterien für Ihre persönliche Verkehrsmittelwahl auf dem Arbeitsweg -  Bitte kreuzen Sie in jeder Zeile ein Kästchen an. [Flexibilität während der Dienstzeit (z.B. Besprechungen)] | wichtig/ eher wichtig/ unwichtig/ eher unwichtig |
| Wie wichtig sind Ihnen die folgenden Kriterien für Ihre persönliche Verkehrsmittelwahl auf dem Arbeitsweg -  Bitte kreuzen Sie in jeder Zeile ein Kästchen an. [Sonstiges] | wichtig/ eher wichtig/ unwichtig/ eher unwichtig |
| Bitte beschreiben Sie kurz "Sonstiges": |  |
| Wie viele Wege (Hin- und Retourweg ist 1 Weg) legen Sie außer dem Arbeitsweg üblicherweise an einem Werktag zurück | keinen Weg/ 1 Weg/ 2 Wege/ mehr als 2 Wege |
| Welchen Wegzweck hatte dieser erste zusätzliche typische private Weg | Private Erledigung/ Privater Besuch/ Einkauf/ Sonstige Freizeit/ "Bringen/Holen/ Begleiten von Personen" |
| Welchen Wegzweck hatte dieser erste zusätzliche typische private Weg - [Sonstiges] |  |
| Mit welchem Verkehrsmittel bzw. mit welchen Verkehrsmitteln haben Sie diesen ersten privaten Weg zurückgelegt -  Bitte alle benutzten Verkehrsmittel angeben! [zu Fuß] | ja/nein |
| Mit welchem Verkehrsmittel bzw. mit welchen Verkehrsmitteln haben Sie diesen ersten privaten Weg zurückgelegt -  Bitte alle benutzten Verkehrsmittel angeben! [Rad] | ja/nein |
| Mit welchem Verkehrsmittel bzw. mit welchen Verkehrsmitteln haben Sie diesen ersten privaten Weg zurückgelegt -  Bitte alle benutzten Verkehrsmittel angeben! [E-Bike] | ja/nein |
| Mit welchem Verkehrsmittel bzw. mit welchen Verkehrsmitteln haben Sie diesen ersten privaten Weg zurückgelegt -  Bitte alle benutzten Verkehrsmittel angeben! [Moped / Motorrad] | ja/nein |
| Mit welchem Verkehrsmittel bzw. mit welchen Verkehrsmitteln haben Sie diesen ersten privaten Weg zurückgelegt -  Bitte alle benutzten Verkehrsmittel angeben! [Pkw als FahrerIn] | ja/nein |
| Mit welchem Verkehrsmittel bzw. mit welchen Verkehrsmitteln haben Sie diesen ersten privaten Weg zurückgelegt -  Bitte alle benutzten Verkehrsmittel angeben! [Pkw als MitfahrerIn] | ja/nein |
| Mit welchem Verkehrsmittel bzw. mit welchen Verkehrsmitteln haben Sie diesen ersten privaten Weg zurückgelegt -  Bitte alle benutzten Verkehrsmittel angeben! [O-Bus] | ja/nein |
| Mit welchem Verkehrsmittel bzw. mit welchen Verkehrsmitteln haben Sie diesen ersten privaten Weg zurückgelegt -  Bitte alle benutzten Verkehrsmittel angeben! [Regionalbus] | ja/nein |
| Mit welchem Verkehrsmittel bzw. mit welchen Verkehrsmitteln haben Sie diesen ersten privaten Weg zurückgelegt -  Bitte alle benutzten Verkehrsmittel angeben! [Bahn (inkl. Schnell-/Lokalbahn)] | ja/nein |
| Mit welchem Verkehrsmittel bzw. mit welchen Verkehrsmitteln haben Sie diesen ersten privaten Weg zurückgelegt -  Bitte alle benutzten Verkehrsmittel angeben! [Sonstiges (z.B. Taxi)] | ja/nein |
| Wie lang ist die Strecke für diesen ersten typischen privaten Weg - [ca. in Kilometer ...] |  |
| Welchen Wegzweck hatte dieser zusätzliche zweite typische private Weg | Private Erledigung/ Privater Besuch/ Einkauf/ Sonstige Freizeit/ "Bringen/Holen/ Begleiten von Personen" |
| Welchen Wegzweck hatte dieser zusätzliche zweite typische private Weg - [Sonstiges] |  |
| Mit welchem Verkehrsmittel bzw. mit welchen Verkehrsmitteln haben Sie diesen zweiten privaten Weg zurückgelegt -  Bitte alle benutzten Verkehrsmittel angeben! [zu Fuß] | ja/nein |
| Mit welchem Verkehrsmittel bzw. mit welchen Verkehrsmitteln haben Sie diesen zweiten privaten Weg zurückgelegt -  Bitte alle benutzten Verkehrsmittel angeben! [Rad] | ja/nein |
| Mit welchem Verkehrsmittel bzw. mit welchen Verkehrsmitteln haben Sie diesen zweiten privaten Weg zurückgelegt -  Bitte alle benutzten Verkehrsmittel angeben! [E-Bike] | ja/nein |
| Mit welchem Verkehrsmittel bzw. mit welchen Verkehrsmitteln haben Sie diesen zweiten privaten Weg zurückgelegt -  Bitte alle benutzten Verkehrsmittel angeben! [Moped / Motorrad] | ja/nein |
| Mit welchem Verkehrsmittel bzw. mit welchen Verkehrsmitteln haben Sie diesen zweiten privaten Weg zurückgelegt -  Bitte alle benutzten Verkehrsmittel angeben! [Pkw als FahrerIn] | ja/nein |
| Mit welchem Verkehrsmittel bzw. mit welchen Verkehrsmitteln haben Sie diesen zweiten privaten Weg zurückgelegt -  Bitte alle benutzten Verkehrsmittel angeben! [Pkw als MitfahrerIn] | ja/nein |
| Mit welchem Verkehrsmittel bzw. mit welchen Verkehrsmitteln haben Sie diesen zweiten privaten Weg zurückgelegt -  Bitte alle benutzten Verkehrsmittel angeben! [O-Bus] | ja/nein |
| Mit welchem Verkehrsmittel bzw. mit welchen Verkehrsmitteln haben Sie diesen zweiten privaten Weg zurückgelegt -  Bitte alle benutzten Verkehrsmittel angeben! [Regionalbus] | ja/nein |
| Mit welchem Verkehrsmittel bzw. mit welchen Verkehrsmitteln haben Sie diesen zweiten privaten Weg zurückgelegt -  Bitte alle benutzten Verkehrsmittel angeben! [Bahn (inkl. Schnell-/Lokalbahn)] | ja/nein |
| Mit welchem Verkehrsmittel bzw. mit welchen Verkehrsmitteln haben Sie diesen zweiten privaten Weg zurückgelegt -  Bitte alle benutzten Verkehrsmittel angeben! [Sonstiges (z.B. Taxi)] | ja/nein |
| Wie lang ist die Strecke für diesen zweiten typischen privaten Weg - [ca. in Kilometer ...] |  |
| Welchen Wegzweck hatte dieser zusätzliche dritte typische private Weg | Private Erledigung/ Privater Besuch/ Einkauf/ Sonstige Freizeit/ "Bringen/Holen/ Begleiten von Personen" |
| Welchen Wegzweck hatte dieser zusätzliche dritte typische private Weg - [Sonstiges] |  |
| Mit welchem Verkehrsmittel bzw. mit welchen Verkehrsmitteln haben Sie diesen dritten privaten Weg zurückgelegt -  Bitte alle benutzten Verkehrsmittel angeben! [zu Fuß] | ja/nein |
| Mit welchem Verkehrsmittel bzw. mit welchen Verkehrsmitteln haben Sie diesen dritten privaten Weg zurückgelegt -  Bitte alle benutzten Verkehrsmittel angeben! [Rad] | ja/nein |
| Mit welchem Verkehrsmittel bzw. mit welchen Verkehrsmitteln haben Sie diesen dritten privaten Weg zurückgelegt -  Bitte alle benutzten Verkehrsmittel angeben! [E-Bike] | ja/nein |
| Mit welchem Verkehrsmittel bzw. mit welchen Verkehrsmitteln haben Sie diesen dritten privaten Weg zurückgelegt -  Bitte alle benutzten Verkehrsmittel angeben! [Moped / Motorrad] | ja/nein |
| Mit welchem Verkehrsmittel bzw. mit welchen Verkehrsmitteln haben Sie diesen dritten privaten Weg zurückgelegt -  Bitte alle benutzten Verkehrsmittel angeben! [Pkw als FahrerIn] | ja/nein |
| Mit welchem Verkehrsmittel bzw. mit welchen Verkehrsmitteln haben Sie diesen dritten privaten Weg zurückgelegt -  Bitte alle benutzten Verkehrsmittel angeben! [Pkw als MitfahrerIn] | ja/nein |
| Mit welchem Verkehrsmittel bzw. mit welchen Verkehrsmitteln haben Sie diesen dritten privaten Weg zurückgelegt -  Bitte alle benutzten Verkehrsmittel angeben! [O-Bus] | ja/nein |
| Mit welchem Verkehrsmittel bzw. mit welchen Verkehrsmitteln haben Sie diesen dritten privaten Weg zurückgelegt -  Bitte alle benutzten Verkehrsmittel angeben! [Regionalbus] | ja/nein |
| Mit welchem Verkehrsmittel bzw. mit welchen Verkehrsmitteln haben Sie diesen dritten privaten Weg zurückgelegt -  Bitte alle benutzten Verkehrsmittel angeben! [Bahn (inkl. Schnell-/Lokalbahn)] | ja/nein |
| Mit welchem Verkehrsmittel bzw. mit welchen Verkehrsmitteln haben Sie diesen dritten privaten Weg zurückgelegt -  Bitte alle benutzten Verkehrsmittel angeben! [Sonstiges (z.B. Taxi)] | ja/nein |
| Wie lang ist die Strecke für diesen dritten typischen privaten Weg - [ca. in Kilometer ...] |  |
